# Interventions to prevent or treat childhood obesity in Māori & Pacific Islanders: a systematic review

**DOI:** 10.1186/s12889-020-08848-6

**Published:** 2020-05-19

**Authors:** Robyn Littlewood, Oliver J. Canfell, Jacqueline L. Walker

**Affiliations:** 1 Health and Wellbeing Queensland, Queensland Government, The State of Queensland, 139 Coronation Drive, Milton Green, Milton, QLD 4064 Australia; 2 School of Human Movement and Nutrition Sciences, Faculty of Health and Behavioural Sciences, The University of Queensland, St Lucia, QLD 4067 Australia; 3 Children’s Health Queensland Hospital and Health Service, Department of Health, Queensland Government, South Brisbane, QLD 4101 Australia

**Keywords:** Oceanic ancestry group, Child, Adolescent, Obesity, Interventions

## Abstract

**Background:**

Māori and Pacific Islander people are a priority population originating from Australasia. Māori and Pacific Islander children exhibit greater risk of obesity and associated morbidities compared to children of other descent, secondary to unique cultural practices and socioeconomic disadvantage. Despite these known risk factors, there is limited synthesised evidence for preventing and treating childhood obesity in this unique population. The objective of this systematic review was to identify and evaluate global prevention or treatment interventions for overweight or obesity that targeted Māori and Pacific Islander children and adolescents (aged 2–17 years).

**Methods:**

The Preferred Reporting Items for Systematic Reviews and Meta-Analyses (PRISMA) guidelines were followed. The databases PubMed, EMBASE, Scopus, Web of Science and CINAHL were searched from inception to August 2018. Study quality and risk of bias was assessed using a modified Downs and Black Quality Checklist for Health Care Intervention Studies. Studies were included if RCT/intervention/case control/ or prevention study designs. The study group was defined under the search term ‘Oceanic Ancestry Group’.

**Results:**

Of the initial 94 articles identified, six were included describing two prevention and three treatment interventions. Interventions were heterogenous in setting, design, length and outcomes. Four interventions were implemented in New Zealand. Most studies were of ‘fair’ quality. One study recruited an exclusive population of Māori and Pacific Islander participants. In the five studies that recruited mixed populations, one performed sub-group analysis on Māori and Pacific Islander participants. No study reported an improvement in anthropometric outcomes post-intervention in complete or sub-group analysis. Improvements in cardiometabolic or psychological secondary outcomes were inconsistent across all studies.

**Conclusions:**

There is a lack of evidence to recommend specific intervention characteristics to optimise obesity prevention or treatment outcomes for Māori and Pacific Islander children. Future research requires greater consideration of cultural values and beliefs, community engagement, exclusive targeting of Māori and Pacific Islander children and families, and sub-group analyses for mixed-population studies. Incorporating co-design principles during study design and implementation can maximise the cultural specificity of interventions and may contribute to improved health and weight-related outcomes for this at-risk, priority population.

**Trial registration:**

PROSPERO CRD42019121790 (26 March 2019).

## Introduction

Overweight and obesity (overweight/obesity) is one of the greatest global public health challenges of modern society. In Australia, obesity is defined as a National Health Priority Area [1]. Nationally, almost one-quarter (24.9%) of children and adolescents (aged 5–17 years) live with overweight/obesity (17% with overweight and 8.1% with obesity) [2]. There are at-risk priority populations for childhood obesity prevention and treatment within Australia, including Māori (the indigenous people of New Zealand) and Pacific Islander (the collective term for people from different Pacific Island nations) peoples [3]. Briefly, Māori and Pacific Islander people migrated to Australia from four island groups: New Zealand, Micronesia, Melanesia and Polynesia [4]. Māori people began migration to Australia in the late eighteenth century, and migration peaked in the 1960’s secondary to employment and lifestyle opportunities in Australia [5]. In Australia, Māori and Pacific Islanders represent 2.2% (*n* = 518,466) [6] of the population. In New Zealand, 15.6% of the population identify as of Māori heritage and 7.8% of Pacific heritage [7].

Māori and Pacific Islander children of all ages in Australia exhibit a higher estimated prevalence of overweight/obesity at 40.7% (ages 6–11 years) and 49.1% (ages 12–18 years) [8]. There is increased susceptibility to obesity-related comorbidities compared to children of other descent, including type 2 diabetes [4], secondary to continuing generational and socioeconomic disadvantage [9]. In Australia, examples of socioeconomic disadvantage in Māori and Pacific Islander peoples include lower weekly income, educational attainment and higher economic stress [4]. Lower community and maternal educational attainment also increases risk of childhood obesity in multiethnic Hawaiian peoples [10]. Similar disparities in childhood obesity prevalence have been observed in New Zealand, with 17% of Māori and 30% of Pacific Islander children identified with obesity in 2017/18, compared to the national average of 12% [11].

Identifying and evaluating global interventions to treat overweight/obesity in children/adolescents continues to be a priority research area. Conclusions from recent systematic reviews highlight multicomponent dietary, physical activity and behaviour-change interventions as the most effective [12, 13]. Despite this, transferability of prevention and treatment interventions to multiethnic priority populations requires further contextualisation. Success in childhood obesity prevention and treatment in Māori and Pacific Islander populations is dependent on designing and implementing interventions that are culturally-tailored [14]. Cultural tailoring is the use of unique population cultural attributes to develop and implement strategies for change [15]. Significant risk factors for obesity and barriers to its prevention and treatment exist across multiple domains specific to Māori and Pacific Islander social determinants of health, including: a higher risk of socioeconomic disadvantage; limited access to and understanding of healthcare services; very low health literacy; and low levels of health-seeking behaviour [4].

While the authors acknowledge there is great diversity in cultural values and beliefs in Māori and Pacific Islander peoples, there are similarities that require consideration when targeting childhood obesity. The key to achieving good health for Māori peoples, as reported by this population, is a balance between mental, physical, family/social, and spiritual domains [5]. Family and social aspects of life contribute significantly to overall wellbeing [5, 16]. Māori and Pacific Islander peoples are inclined to direct help-seeking behaviour towards other members of their communities and thus health services are often neglected in favour of familial care and support [5]. Sharing food with family and guests, particularly certain cultural foods, such as taro and coconut, is also a strongly integrated socio-cultural tradition [16, 17]. Māori and Pacific Islander peoples embed value in people with larger stature and bodies, associating this with enhanced health, higher beauty and greater wealth [16]. Additionally, religion is a significant component of Māori and Pacific Islander culture, with Catholicism integrated heavily into families and various cultural practices [5, 17].

Despite the cultural, socioeconomic and overweight/obesity prevalence disparities in Māori and Pacific Islander peoples, no known evidence has been systematically collated for childhood overweight/obesity prevention and treatment interventions in this population. The aim of this review is to identify and evaluate global studies reporting interventions to prevent or treat childhood and/or adolescent overweight/obesity in the unique population of Māori and Pacific Islander peoples. Results of this review will create a unique evidence-base that may be used to guide future culturally tailored childhood obesity prevention and treatment interventions to maximise outcomes for childhood overweight/obesity in Māori and Pacific Islander populations.

## Methods

The protocol for this systematic review was registered with PROSPERO in the early stages of commencement (CRD42019121790, available at http://www.crd.york.ac.uk/PROSPERO/display_record.php?ID=CRD42019121790). The PRISMA (Preferred Reporting Items for Systematic Reviews and Meta-Analyses) guidelines were followed in preparing this review (see Additional file 1 for PRISMA checklist).

### Search strategy and identification of included articles

The databases PUBMED, EMBASE, SCOPUS, Web of Science and CINAHL were searched from inception to August, 2018. Keywords were identified via discussions with authors and a research librarian (see Additional file 2 for the full search strategy). A primary term used to identify populations of Māori and Pacific Islander descent was “Oceanic Ancestry Group”, defined as: “Individuals whose ancestral origins are in the islands of the central and South Pacific, including Micronesia, Melanesia, Polynesia, and traditionally Australasia” [18].

The search strategy was developed and implemented using the PICO (Population, Intervention, Comparison, Outcome) format:

1. *Population* – Māori and Pacific Islander children or adolescents aged 2–17 years;
2. *Interventions* - e.g. a program, health service, strategy or initiative to prevent or treat overweight/obesity;
3. *Comparison* – no program, health service, strategy or initiative; and
4. *Outcomes* – anthropometric (weight, BMI, body composition), cardiometabolic, psychological, behavioural.

The search strategy was optimised in EMBASE and then translated to all other databases. One reviewer (RL) screened all articles, then reviewed abstracts of relevant articles to identify those appropriate for full-text review. Two reviewers (RL and OJC) conducted full-text review of identified articles, based on relevant inclusion and exclusion criteria. The reference lists of all articles identified for full-text review were also screened for potential additional articles.

### Inclusion and exclusion criteria

Studies were included if they described the implementation of a prevention or treatment intervention for overweight/obesity in Māori or Pacific Islander children and/or adolescents (aged 2–17 years). Studies were required to meet the following inclusion criteria:

(i) RCT, pre-post intervention, case-control or prevention study design; and
(ii) Subjects or sub-group of subjects were defined under the ‘Oceanic Ancestry Group’, including Māori & Pacific Islander; and
(iii) Subjects were children or adolescents aged 2–17 years; and
(iv) Intervention, health service, program, strategy, or initiative was to prevent or treat overweight or obesity; and
(v) For treatment interventions, included pre-post anthropometric (i.e. weight, BMI and/or body composition) measures as an outcome, with or without cardiometabolic, psychological or behavioural outcomes; and
(vi) For prevention interventions, included a pre-post nutrition and/or obesity-related outcome measure (e.g. diet, screen time, anthropometry, parental feeding practices, sleep, health-related quality of life [HR-QOL], physical activity [PA], health literacy/knowledge).

Studies were excluded if they met the following exclusion criteria:

(i) Study design did not pertain to the prevention or treatment of overweight/obesity; or
(ii) Studies not published as full-text articles.

No restrictions were placed on language of the published article.

### Data extraction and synthesis

One reviewer (OJC) extracted the following information from each included article for qualitative synthesis: country of intervention; study design; participant characteristics (age range, sex distribution, ethnic distribution); recruitment strategies; details of randomization (if any); intervention setting, description and dose; participant retention; control group characteristics; length of follow-up; outcome measure/s; appropriateness of statistical tests; details of sub-group analysis; and main findings at each follow-up timepoint. Initial data extraction was cross-checked by a second reviewer (RL) for accuracy, with any discrepancies resolved by discussion. Due to the heterogeneity between study design and outcome measures for prevention and treatment interventions, a quantitative meta-analysis approach was deemed inappropriate and a qualitative synthesis of results was conducted.

### Quality assessment and risk of bias

Study quality and risk of bias was assessed independently by two reviewers (RL and OJC) using the Downs and Black Quality Checklist for Health Care Intervention Studies [19]. The checklist assesses quality and risk of bias of randomized and non-randomized studies using five domains: reporting; external validity; bias; confounding; and power [19]. A previously used [20] modified version of the checklist was used for simplicity and clarity: instead of calculating a range of potential study powers in Item 27, we determined if each study performed a power calculation (1 = yes, 0 = no), thus making 28 the highest possible score for the checklist. Downs and Black scores were assigned to the following score ranges, according to Hooper et al. [21]: excellent (26–28); good (20–25); fair (15–19); and poor (≤14). Any discrepancies in the article screening, data extraction and synthesis, or quality and risk of bias assessment phases were discussed and resolved with a third reviewer (JLW).

## Results

### Identification of included articles

**Fig. 1:**
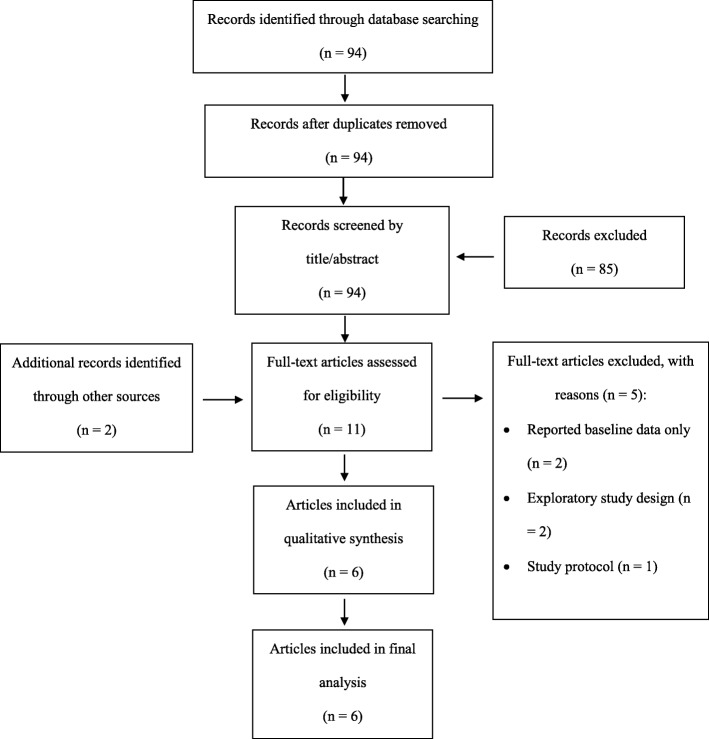
PRISMA flow diagram

A total of 94 articles were identified from the initial search (see Figure 1 for PRISMA flow diagram for the present systematic review). Following duplicate removal and title/abstract screening, 9 articles remained. An additional two articles were identified via reference list scoping of identified full-text articles. Of these 11 articles, six met the inclusion criteria and were included for qualitative synthesis in the present systematic review [22–27]. The six articles described outcomes from five independent programs or initiatives. While two articles describe results from a single program [25, 26], both were included in this review as they assessed different outcome measures and were heterogenous in follow-up time.

### Quality assessment and risk of bias

Table 1 describes the quality assessment and risk of bias results for each study. According to the modified Downs and Black checklist, four articles were assessed to be of ‘fair’ quality [23–25, 27] and two articles of ‘good’ quality [22, 26]. The strongest scoring article [25] (23 points of a possible 28) exceeded in their recruitment of subjects representative of the source population, blinding of participant schools receiving the intervention, and the details provided regarding randomization techniques. See Additional file 3 for completed Downs and Black checklists for each included article.

**Table 1:** Study quality assessment results completed according to the Downs and Black checklist for randomised and non-randomised healthcare interventions [19]

Authors & Country	Design	Downs and Black score^a^	Study quality
Anderson, C.Y et al. (2017) [22] (New Zealand)	RCT (Treatment)	20	Good
Chansavang, Y. et al. (2015) [23] (New Zealand)	Pre/post mixed methods (Treatment)	16	Fair
Gittelsohn, J. et al. (2010) [24] (USA - Hawaii)	Pre/post with control (Prevention)	17	Fair
Maddison, R. et al. (2014) [27] (New Zealand)	RCT (Treatment)	19	Fair
Rush, E. et al. (2012) [26] (New Zealand)	RCT (Prevention)	23	Good
Rush, R. et al. (2014) [25] (New Zealand)	RCT (Prevention)	19	Fair

*RCT* Randomised controlled trial

^a^Out of a possible 28. Score ranges: excellent (26–28); good (20–25); fair (15–19); and poor (≤14)

### Study participants

The total sample size for each study ranged from 18 [23] to 6629 [25] (mean: 1431). Programs targeted: both children and adolescents (5–16 y) (*n* = 1) [22]; younger (5–7 years [26], and 6–8 years [25]) and older (10–12 y [26], and 9–11 y [25]) children separately, but within the same school program (*n* = 2); older children only (mean age 9.8 y [24], and aged 9–12 y [27]) (*n =* 2); and adolescents only (mean age 16.3 y [23]) (*n =* 1). A higher percentage of female participants was reported in three studies [23, 25, 26], with one study reporting equal sex distribution [24].

Only one program (Chansavang et al. [23]), implemented in New Zealand, recruited an exclusive Māori and Pacific Islander population; however the total sample size was 18. All remaining programs reported a combination of Māori, Pacific Islander and those of other descent (typically New Zealand-European [NZE]) participating in the intervention arm of each program. Of the remaining programs implemented in New Zealand [22, 25–27], two (Whānau Pakari [22] and SWITCH [27]) reported a higher recruited proportion of Māori and/or Pacific Islander participants than those of other descent (45% vs. 41% [22], and 66% vs. 34% [27], respectively). Project Energize reported a higher proportion of NZE participants compared to Māori and Pacific Islanders at the first follow-up timepoint of 2 years [26] in both the younger (67% vs. 23%) and older (60% vs. 33%) participants, and in the second follow-up timepoint of 5 years [25] in the younger (54.3% vs. 41.9%) and older (53.7% vs. 42.7%) participants. At the first follow-up timepoint [26], only participants who identified as Māori were reported; there were no prevalence statistics for Pacific Islander participants. In the Hawaii-based HFH program described by Gittelsohn et al. [24], 64% of participants who undertook pre-post questionnaires for the HFH program self-identified as Native Hawaiian or other Pacific Islander.

### Study characteristics

Table 2 presents the study design, participant and retention rate, intervention and outcome characteristics of each study. Four of the five programs were implemented in New Zealand [22, 23, 25–27] and the remaining program was implemented in Hawaii, USA [24]. Three programs were RCTs [22, 25–27], one of which was participant-blinded [25, 26], and all provided details of a non-biased randomization technique that was used. The remaining two programs were of pre-post intervention design [23, 24], one of which incorporated a non-blinded, non-randomized control group [24]. One pre-post intervention program employed a mixed-methods design, integrating qualitative comments from participants on program feasibility as an outcome measure [23].

**Table 2:** Characteristics of included studies of prevention or treatment interventions for Māori and Pacific Islander children and adolescents

Authors & Country	Design	Participants and retention	Intervention Characteristics			Control	Outcome Measure/s	Main Findings
			Setting	Description	Dose			
Anderson, C.Y et al. [22] (2017) (NZ)	RCT (Treatment)	*n =* 103 (I: *n =* 69; C: *n =* 69); aged 5–16 y with overweight/ obesity and comorbidities; 49% female; 45% Māori Retention: 69% (31% dropout)	Community sporting venues (intervention); Home (assessments)	*“Whānau Pakari Program”* Multidisciplinary program, delivered by a physical activity coordinator, dietitian, and psychologist. Sessions focused on an introduction to sports, making sustainable healthy lifestyle change and dietary education.	12-month, weekly group sessions. 6- and 12-month follow-up with home visits, assessments, and advice	Physical assessments and advice bi-annually for 2 years	(1) Anthropometry: BMI SDS change, baseline − 12 mo. (2) Psychological: quality of life (HR-QOL); psychological characteristics (CBCL); Cardiometabolic: PA (steps/day; PA intensity); CV fitness^a^; glycated hemoglobin; fasting insulin; Behavioural: screen time	No difference in BMI SDS reduction after 12 mo in Māori participants. CV fitness and HR-QOL sig. Improved in Māori participants. Attendance of ≥70% sessions sig. Increased BMI SDS reduction, CV fitness, parent HR-QOL and CBCL score.
Chansavang, Y. et al. [23] (2015) (NZ)	Pre/post mixed methods (Treatment)	*n* = 18; “less-active adolescents”, mean age 16.3 y; 78% female; 72% Pacific; 28% Māori Retention: 89% (11% dropout)	Recreation Centre (after-school)	Group-based exercise and lifestyle intervention program. Sessions focused on a variety of physical activities, with dietary and lifestyle education delivered post-session. Text message support was provided, containing health-related quotes.	6-week, 3 × 1.5 h per week sessions; follow-up at intervention conclusion	None	(1) Cardiometabolic: VO_2_max; insulin resistance (2) Cardiometabolic: glycated hemoglobin; fasting plasma glucose; fasting lipid profile; PA levels (IPAQ); Anthropometry: BMI, waist circumference; Behavioural (qualitative): session attendance; comments on program feasibility	Significant improvements in VO_2_max, systolic BP, weekly vigorous and moderate PA; however, waist circumference sig. Increased. No change in BMI or weight. Feasibility comments were positive, related to sport participation and helpfulness of texts.
Gittelsohn, J. et al. [24] (2010) (USA - Hawaii)	Pre/post with control (Prevention)	*n =* 117 (I: *n =* 64; C: *n =* 53) child-caregiver pairs; mean child age 9.8 y; 65% Native Hawaiian or Pacific Islander; 50% female (child); 95% female (caregiver) Retention: 67% (33% dropout)	Five stores in two communities in Oahu and the Big Island. Population size: Oahu (*n =* 10,506); Big Island (*n =* 5748)	*“Healthy Foods Hawaii”* Increase availability of healthy foods in community stores. Intervention phases targeted: (1) Healthier beverages; (2) Healthier snacks; (3) Healthier condiments; and (4) Healthier meals. Educational and labelling materials were promoted in-store. Cooking demonstrations performed 4–6 times per phase.	Four phases, 6–8 weeks each, with 1–2 week break intervals.	Two communities on each island with no intervention	(1) Adult caregiver psychosocial factor and food-related behaviours (CIQ); Child psychosocial factors, food-related behaviours and food intake (CCIQ)	Mostly no differences overall; however, significant caregiver improvement in perceiving healthy foods as convenient, and significant child improvement in overall dietary score, particularly water and grain consumption.
Maddison, R. et al. [27] (2014) (NZ)	RCT (Treatment)	*n =* 251 (I: *n =* 127; C: *n =* 124); aged 9–12 years with overweight/ obesity; 43% female; 13% Māori, 53% Pacific Retention: 95% (5% dropout)	Home environment with complementary digital intervention avenues	“*SWITCH*” Reducing all leisure-based screen-time activities in the home. Three elements offered to families: (1) Behaviour change strategies; (2) Budgeting media time; (3) Activity pack for children.	20 weeks, initial face-to-face, then monthly digital resources. Follow-up at 24 week post-randomisation (4 weeks post-intervention)	Usual behaviour	(1) Anthropometry: BMI z-score (2) Anthropometry: BMI, weight (kg), WC, %BF; Cardiometabolic: PA frequency & intensity; Behavioural: total sedentary time (mins), sleep, dietary intake, enjoyment of PA and sedentary behaviour	No significant differences.
Rush, E. et al. [26] (2012) (NZ)	RCT (Prevention)	*n =* 926 (I: *n* = 492; C: *n* = 434); aged 5–7 y; 51% female; 23% Māori *n =* 446 (I: *n =* 200; C: *n =* 226); aged 10–12 y; 51% female; 33% Māori Retention: Aged 5-7y - 80% (20% dropout) Aged 10-12y − 57% (43% dropout)	124 primary schools	“*Project Energize”* Assignment of a dedicated healthy lifestyle champion - “Energizer” - to each school. Energizers were “agents of change” and integrated physical activity, healthy eating and educational initiatives into daily class activities. Parental nutrition education sessions were delivered.	2 years, no specific dose. Assessments at baseline and 2 years.	Schools - no intervention with no restrictions on self-directed initiatives	(1) Anthropometry: body composition (BMI; %BF); Cardiometabolic: BP;	No significant differences in Māori population.
Rush, R. et al. [25] (2014) (NZ)	RCT (Prevention)	*n* = 2959 (I: *n* = 2474; C: *n =* 485); aged 6–8 y; 52% female; 36% Māori *n* = 3670 (I: *n* = 2330; C: *n =* 1340); aged 9-11y; 54% female; 37% Māori Retention (number of schools): 82% (18% dropout)	193 primary schools	As above	Months (*n*) of engagement with each school	Historical comparison with 2012 RCT control group [26]	(1) Cardiometabolic: BP; CV fitness^a^; Anthropometry: body composition (BMI; %BF)	Overweight/obesity prevalence 31 and 15% lower in younger and older “Energized” children compared to historical comparison, respectively. BMI lower by 3 and 2.4%, respectively. Physical fitness also higher.

*NZ* New Zealand, *RCT* Randomised controlled trial, *BMI* Body mass index, *SDS* Standard deviation score, *HR-QOL* Health-related quality of life, *CBCL* Child behaviour checklist, *PA* Physical activity, *CV* Cardiovascular, *IPAQ* International Physical Activity Questionnaires, *BP* Blood pressure, *CIQ* Caregiver Impact Questionnaire, *CCIQ* Child Customer Impact Questionnaire, *SWITCH* Screen-Time Weight-loss Intervention Targeting Children at Home, *BF* Body fat

^a^Assessed by a 550-m walk/run time trial

#### Prevention

Two programs (‘Healthy Foods Hawaii’ [HFH] [24] and ‘Project Energize’ [25, 26]) were classified as prevention interventions [24–26]. HFH targeted the local food environment in low-income, multiethnic communities on the islands of Oahu and Big Island in Hawaii, USA [24]. Local health center patient databases were used to randomly recruit participants. Four phases were implemented over 9–11 months in five food stores focusing on: healthier beverages; healthier snacks; healthier condiments; and healthier meals. Educational materials to promote healthier choices included: posters; display; and shelf-labels, with a cooking demonstration held in each store during each phase.

Project Energize [25, 26] was a widespread school-based overweight/obesity prevention intervention delivered in the Waikato region of New Zealand, targeting children aged 5 and 10 years at the time of commencement. Two included articles described outcomes from Project Energize – the first reporting pre-post outcomes from a 2-year RCT [26], and the second reporting results in a new cohort (2011), using historical results from the control cohort 5-years earlier (2006) as a comparison [25]. In the 2-year RCT, schools were identified from a representative national list, randomised and subsequently approached for study inclusion. In the 2011 cohort, schools were invited to participate if actively engaged in Project Energize for at least 18 months. The intervention was consistent between articles: a group of teachers or graduates in exercise and nutrition, called “Energizer’s”, were responsible for the unique development and delivery of each school’s intervention. Energizer’s coordinated physical exercise and games during and outside of classes, and provided support for each school to implement healthy eating initiatives, including making modifications to canteen choices and providing incentives to purchase healthier options. Nutrition educational resources were delivered in weekly newsletters to children and parents, and parents were offered three practical nutrition classes throughout the intervention.

#### Treatment

Three programs were classified as treatment interventions (‘Whānau Pakari Program’, ‘SWITCH’, and Chansavang et al. [22, 23, 27]), as program participants were recruited based on classification with overweight/obesity [22, 27] or if defined as “less-active” adolescents [23] (participating in less than two exercise sessions per week), all of whom were classified with overweight/obesity.

The Whānau Pakari treatment intervention was a 12-month multidisciplinary program delivered weekly by a physical activity coordinator, dietitian and psychologist at community sporting venues to children with overweight/obesity (aged 5–16 y; 45% Māori) [22]. Children were recruited from a clinical, community-based service for all children and adolescents with obesity, with a focus on recruiting those of Māori ethnicity and living in more deprived households.

The ‘SWITCH’ trial was a 20-week, family-based RCT targeting screen-time behaviours in children with overweight/obesity (aged 9–12 y; 13% Māori; 53% Pacific Islander). Recruitment avenues included schools, community centres, churches, primary healthcare organisations and word of mouth [27]. Primary caregivers were provided with relevant education materials at intervention commencement, as well as a screen-time monitoring device – “Time Machine”. No further face-to-face contact was provided for the duration of the intervention; however, newsletters with further screen-time reduction strategies were disseminated to caregivers monthly, and an activity pack was given to child participants that provided non-screen-time activity options.

In the study of Chansavang et al. [23], Māori & Pacific Islander adolescents with overweight/obesity (*n = 1*8; aged 16y) were recruited from a secondary school in a low socioeconomic area in a 6-week after-school based exercise and lifestyle intervention. Sessions of 1.5-h were delivered three times weekly, incorporating a variety of physical activities of moderate to high intensity, with “Healthy snacks” and healthy eating education provided to participants after each session. Motivational text message support was also provided throughout the intervention.

### Intervention effectiveness

#### Prevention

Table 3 describes the effectiveness of all prevention and treatment interventions on overall participant populations within studies included in the present review, including results of sub-group analyses for Māori and Pacific Islander participants. Neither prevention intervention (HFH and Project Energize) reported significant post-intervention improvements in anthropometric indicators of weight. Only HFH [24] reported a statistically significant improvement in outcome measures, which were psychological (parent food knowledge) and behavioural (overall child dietary intake). No significant changes were observed in child and caregiver psychosocial food-related factors, or caregiver food-related behaviours. No sub-group analysis results for Māori and Pacific Islander participants were reported in the HFH program.

**Table 3:** Comparison of the effectiveness of prevention and treatment interventions on outcomes of interest in overall participants and the sub-group of Māori and Pacific Islander participants

Authors & Country	Study type	Sub-group analysis of MPI participants	Effectiveness on intervention outcomes			
			Improved anthropometry	Improved cardiometabolic	Improved psychological	Improved behavioural
Anderson, C.Y et al. [22] (2017) (NZ)	Treatment	Yes (cardiometabolic only)	**×** (BMI, WC)^a^	**×** (HbA_1c_, fasting insulin, CV fitness^b^, PA levels/intensity**)** **✓** CV fitness^a^	✓ (HR-QOL, overall psychological profile)^a^	**×** (Screen time)
Chansavang, Y. et al. [23] (2015) (NZ)	Treatment	Participants were exclusively of MPI descent	**×** (BMI, WC)^a^	✓ (VO_2_max, BP, HbA_1c_)^a^ **×** (fasting insulin)^a^	–	–
Gittelsohn, J. et al. [24] (2010) (USA - Hawaii)	Prevention	No	–	–	✓ (Parent food knowledge)	✓ (Overall child dietary intake)
Maddison, R. et al. [27] (2014) (NZ)	Treatment	No	**×** (BMI, %BF, FFM, FM, WC)	–	–	**×** (Screen time, sedentary time, sleep, PA enjoyment, sedentary behaviour enjoyment**)**
Rush, E. et al. [26] (2012) (NZ)	Prevention	Yes	**×** (BMI, %BF)^a^	**×** (BP)^a^	**–**	–
Rush, R. et al. [25] (2014) (NZ)	Prevention	No	NR^c^	NR^‡^	–	–

*MPI* Māori and Pacific Islander, *NZ* New Zealand, *BMI* Body mass index, *WC* Waist circumference, *HbA1c* Glycated haemoglobin, *CV* Cardiovascular, *PA* Physical activity, *BP* Blood pressure, *BF* Body fat, *FFM* Fat-free mass, *FM* Fat mass

^a^Results consistent in sub-group analysis of Māori and Pacific Islander participants

^b^Assessed by a 550-m walk/run time

^c^Not reported: authors did not report effect significance

The 2-year evaluation of Project Energize [26] conducted intervention sub-group analysis on Māori participants, demonstrating increases in anthropometric (BMI SDS, %BF SDS) and cardiometabolic (systolic BP, diastolic BP) outcomes compared to decreases in the same measures for NZE participants; however, these were all to a level of non-significance. In the 5-year evaluation of Project Energize (2011 vs. 2006), compared to the historical comparison and after covariate adjustment, the 2011 children: were slightly younger; had lower weight, BMI and prevalence of overweight/obesity; and did not differ in height [25]. An additional evaluation outcome was cardiometabolic fitness assessed by a 550-m run time, historically compared to data from 664 children from a different region than the original RCT trial (Canterbury vs. Waikato, respectively). Run time was reported as 14 and 11% lower for younger and older children, respectively, who participated in Project Energize, than the historical comparison group from Canterbury [25]. Percentage body fat and systolic and diastolic BP were not assessed in the 5-year Project Energize evaluation study. No sub-group analysis results for Māori and Pacific Islander participants were reported in the 5-year Project Energize historical comparison [25].

#### Treatment

Of the three included studies describing the results of treatment interventions, all assessed pre-post anthropometric indicators of weight [22, 23, 27], two assessed cardiometabolic outcomes [22, 23], two assessed behavioural outcomes [22, 27] and one assessed psychological outcomes [22]. No study reported a significant pre-post intervention improvement in anthropometric measures in Māori and Pacific Islander participants. Both studies measuring behavioural outcomes reported no significant pre-post intervention changes [22, 27].

Chansavang et al. [23] reported a significant post-intervention increase in waist circumference in Māori and Pacific Islander adolescents, yet highlighted meaningful improvements in cardiometabolic indicators (VO_2_max, BP, HbA_1c_ and self-reported exercise levels). Chansavang et al. [23] were the only group to implement a mixed-methods design and approached participants (*n =* 13) for qualitative feedback post-intervention. Participants generally thought positively of the intervention, with physical activity games, novelty of using gym equipment and the provision of food after each session as reported highlights. No qualitative data was provided on possible weaknesses of the intervention or participant-identified improvements that could be made to the intervention.

The intervention that measured psychological outcomes (Whānau Pakari) reported significant improvements in HR-QOL [22]. Sub-group analysis in the Whānau Pakari intervention found that NZE participants demonstrated a statistically significant improvement in BMI SDS that was not observed in Māori participants; however, Māori participants showed significant improvements in HR-QOL and 550-m run time [22]. Higher attendance (≥70%) in the Whānau Pakari intervention was predictive of greater improvements in BMI SDS and Māori participants were four times less likely than NZE participants to achieve this attendance rate, with only 13% attending ≥70% of sessions.

The SWITCH trial demonstrated no significant changes in any outcome measure, including: anthropometric indicators (BMI, waist circumference, fat-free mass, fat mass, % BF), physical activity level and intensity, sedentary behaviour, or sleep, with sedentary activities still the predominant daily behaviour post-intervention. Sensitivity analysis performed on after-school and weekend sedentary behaviour confirmed these results; no significant changes were reported. Use of the Time Machine was low; 46% of participants reported never using it. No sub-group analysis was performed to assess changes in Māori and Pacific Islander participants.

### Intervention optimisation for Māori and Pacific islander participants

Two included studies specifically mentioned strategies used to optimise their interventions towards Māori and Pacific Islander participants [22, 27]. As part of their Whānau Pakari intervention, Anderson et al. [22] utilised Māori health workers in the community to facilitate recruitment of target families [28]. Additionally, significant consultation was reported with key Māori stakeholders; however, this was limited to the initial set-up phase [28]. No details of specific, culturally-tailored intervention components that arose from cultural consultation were provided, and there was nil mention of further consultation following initial discussions. As part of their SWITCH trial, Maddison et al. [27] mentioned the modification of screen-time-based intervention content to suit Māori and Pacific Islander families; however, no further details were provided and wording was directed towards promoting acceptability, rather than the cultural optimisation of content to elicit maximum intervention impact. The nature of content changes remained unclear and there was no mention of community or consumer consultation in designing the intervention. The remaining included studies [23–26] did not report implementation of intervention co-design with Māori and Pacific Islanders, or community and consumer consultation in an attempt to culturally optimise their interventions.

## Discussion

### Main findings

This is the first known review to systematically identify, appraise and evaluate interventions to prevent or treat overweight/obesity in the unique, at-risk priority population of Māori and Pacific Islander children/adolescents. A total of six studies were identified, describing five separate prevention and treatment interventions, that targeted Māori and Pacific Islander children/adolescents, with only one [23] exclusively targeting this population. Interventions were generally heterogenous in their development, design, size, length and outcomes. No study reported a significant improvement in weight-related anthropometric indicators post-intervention in Māori and Pacific Islander participants. Improvements in cardiometabolic outcomes for Māori and Pacific Islander participants were observed in two treatment interventions [22, 23], one with an exclusive population of Māori and Pacific Islanders [23] and the other after performing sub-group analysis [22], an effect not observed in NZE participants in the latter. Both studies [22, 24] assessing psychological outcomes reported significant improvements, which were retained in sub-group analysis of Māori and Pacific Islander participants in one study [22]. Two of three studies assessing behavioural secondary outcomes reported no significant changes [22, 27], with the remaining study highlighting positive changes in child dietary intake [24]; however, none of these studies performed sub-group analysis for Māori and Pacific Islander participants.

### Cultural optimisation of interventions

Interventions that are contextualised to culture, ethnicity and socioeconomic status have higher retention and success [16]. Consistent with Māori and Pacific Islander cultural values, higher retention of these children in obesity interventions is strongly linked to family and group participation and shared parent-child learning experiences [29]. Co-design in healthcare is a participatory, experience-based activity that is a collaboration between the target population (e.g. patients, community members, specific cultural groups) and healthcare professionals within the system to ensure the outcome is representative of participant needs and expectations [3, 30]. Co-design empowers its target population by facilitating shared and equal decision-making, and promotes enhanced satisfaction with the designed intervention, as well as improved health outcomes [31]. Only one included study [22] described a consultation phase with Māori stakeholders to assist with intervention design; however, there was no specific mention of co-design methodologies, or the results of this consultation. Despite this consultation, the authors still acknowledged greater incorporation of Māori culture and values was required to address the reported dropout rate (31%) by Māori and Pacific Islander participants [22].

A possible explanation for the null findings in intervention effectiveness for weight-related outcomes across all studies, as well as the low intervention fidelity (dropout rate: 31%) reported by Anderson et al. [22] is the omission of robust co-design methodologies, and thus specificity to a Māori and Pacific Islander population. Well-established cultural values, beliefs and traditions of Māori and Pacific Islander peoples, such as: attributing good health to more than physical attributes; using traditional food and feasts as a focal point for social gatherings; and placing health and beauty value in larger body sizes creates challenges in motivating Māori and Pacific Islander peoples to implement lifestyle, weight-related changes in behaviour [5, 16]. Additionally, similar to other Indigenous populations, such as Aboriginal and Torres Strait Islander peoples, a history of colonisation and oppression has negatively impacted Māori and Pacific Islander peoples’ cultural strength, united voice, collective dignity, and health and wellbeing [5]. Implementing a co-design approach may assist to counter such historical impacts by promoting equity between all stakeholders, stimulating a sense of inclusion, acknowledging and catering for diversity, and creating a medium for openness, respect, and shared purpose [31].

Additionally, five of the six included studies [22, 24–27] described an intervention involving a combination of NZE and Māori and Pacific Islander participants. Tailoring certain intervention components to suit Māori and Pacific Islander participants, as reported in the SWITCH trial [27], when they are still part of a more diverse study population, may adversely generalise the intervention as equally effective and appropriate for all participants. Māori and Pacific Islander peoples are inclined to look within their community for health-related support and guidance; a family-based approach is viewed as essential to taking action in health [5, 14]. Implementing childhood overweight/obesity interventions in exclusively Maori and Pacific Islander populations, as in the study of Chansavang et al. [23], could prove effective in eliciting greater intervention fidelity and outcomes. Tailoring intervention components may also extend beyond adaptations based on language and cultural distinctions. Specifically targeting risk factors for overweight/obesity that are enhanced in Māori and Pacific Islander peoples, such as socioeconomic disadvantage and low health literacy, may prove an effective method of cultural optimisation. For example, incorporating PA solutions into interventions that are financially feasible, or delivering healthy eating education that comprises affordable, yet realistic suggestions to promote behaviour change.

In their recently published article, Verbiest et al. [3] describe the novel and robust implementation of co-design methodology to develop a mobile health (mHealth) behaviour change intervention targeting obesity in an exclusive Māori and Pacific Islander population [3]. As a result of a six-stage process, three key content modules were co-designed (physical activity, family/*whanau* [extended family], and healthy eating), along with 17 culturally-optimised change techniques to facilitate behaviour change, and delivered in separate, individualised Māori and Pacific Islander health apps. The authors concluded that a co-design approach empowered the target population by enabling active, participatory action throughout each phase of design [3].

### Cultural optimisation of system approaches

Whilst standalone trials are essential in building an evidence-base for appropriate childhood overweight/obesity prevention and intervention characteristics in Māori and Pacific Islander peoples, a coordinated whole-of-government, policy-level and healthcare systems response is paramount in contributing to a reduction in prevalence. In response to the growing prevalence of obesity and congruent with the World Health Organization (WHO) Report of the Commission on Ending Childhood Obesity [32], New Zealand’s Ministry of Health developed a nationally coordinated Childhood Obesity Programme, targeting: physical activity, sports and education; health sector; food industry; and public information as broad action sectors to tackle obesity [33]. A focus on Māori and Pacific Islander children was specified; however, strategies for engaging with and culturally-optimising public health interventions for this population remained unclear, specifically in targeting prominent social determinants of health for this population (such as socioeconomic disadvantage, limited engagement with health services) [33]. Prior to the Childhood Obesity Programme, New Zealand launched ‘Healthy Eating – Healthy Action’ in 2003, a national obesity prevention strategy underpinned by the Treaty of Waitangi, the founding document of New Zealand that recognises the cultural partnership between the British Crown and Māori peoples [34]. Priority action areas for Healthy Eating – Healthy Action were conceptualised according to the three principles of: partnership; participation; and protection between Māori peoples and those of non-indigenous heritage, with high-level Māori and Pacific interest groups engaged across implementation and evaluation stages [35, 36]. Integrating a cultural perspective, particularly using co-design, within all public health approaches to childhood obesity prevention and treatment may motivate researchers to engage in similar practices when designing local, health system approaches.

A localised example of a culturally-tailored, integrated health system approach to improving Māori and Pacific Islander child and adolescent health is the ‘Good Start Program’, a statewide, schools-based, nutrition and physical activity initiative embedded within the Queensland Health system, Australia. A qualitative evaluation of its implementation concluded that building a system-wide workforce of Māori and Pacific Islander Health Workers was a significant contributor to enabling high-level community engagement, cultural specificity of interventions, community member satisfaction and trust with the Good Start Program, and ultimately positively shifting Māori and Pacific Islander community members’ attitudes to and knowledge of health [14].

### Strengths and limitations of included articles

There were a number of observed strengths of included studies. Project Energize, the New Zealand schools-based prevention intervention described by Rush et al. [25, 26] in two studies, was extensively integrated within the Waikato region, with the 5-year evaluation reporting 233 engaged schools of a possible 235 (99.1%), reaching over 4000 children [25]. In their recent Cochrane review, Mead et al. [13] called for an increase in qualitative research methods within childhood overweight/obesity treatment intervention study designs to optimise future intervention tailoring. The mixed-methods design described by Chansavang et al. [23] allowed for qualitative intervention feedback to be reported by Māori and Pacific Islander participants, creating a foundation for cultural optimisation and greater applicability to a larger intervention group in future studies. Additionally, Gittelsohn et al. [24] were successful in engaging with key stakeholders across all levels of the local Hawaiian food system as part of their community-wide prevention intervention, including a combination of food producers, distributors and purchasing outlets.

The primary limitation of the current literature is the lack of exclusive recruitment of Māori and Pacific Islander participants and limited sub-group analysis in mixed participant demographics. Descriptions of intervention intensity and intervention fidelity could have been strengthened; this may have contributed to the modesty of reported results, especially anthropometric outcomes. The intervention of Chansavang et al. [23] was implemented in a small sample size (*n* = 18) and the participants of SWITCH trial of Maddison et al. [27] were offered only one initial face-to-face contact with a research assistant. Additionally, the SWITCH trial focused exclusively on screen-time behaviour change, omitting other well-acknowledged components to overweight/obesity prevention, including dietary and physical activity factors [27]. Another potential reason for the reported null anthropometric outcome results is a lack of dietary intervention intensity; physical activity was the primary focus in 3 of 4 programs [22, 25–27] assessing anthropometric outcome measures. The 5-year evaluation of Project Energize [25] was conducted without a contemporaneous control group and assessed different outcome measures between the 2-year [26] and 5-year post-intervention evaluations. The HFH prevention intervention of Gittelsohn et al. [24] analysed local community impact within a sub-group of child-caregiver pairs (*n =* 116), which may have limited the generalisability of findings to the larger population of Hawaii. Most interventions were described as stand-alone, and there was little evidence of attempted integration within a broader, health system to maximise reach, impact, and sustainability.

### Strengths and limitations of review

The primary strength of this review is its novelty, being the first known to synthesise global evidence of prevention and treatment interventions for the unique, at-risk population of Māori and Pacific Islander children and adolescents. Findings of this review are potentially transferable to priority populations from various developed countries, including the USA and Canada, where young Alaska Native and American Indian children, and First Nations and Inuit peoples, respectively, exhibit high obesity prevalence rates [37]. As an example, in 2013, 32% of Alaska Native children aged 5–12 years were classified as obese [37]. Implementing robust community engagement and intervention co-design processes may be beneficial in tackling childhood obesity within these regions. Additionally, the search strategy was comprehensive and included culturally appropriate search terms. There were no exclusion criteria pertaining to intervention type or setting in an attempt to collate a broad level of evidence. Limitations include a low number of included articles, as well as the modest quality and risk of bias within studies, which limits the ability to develop robust, specific recommendations that are applicable in a wide range of settings for childhood overweight/obesity prevention and treatment in this population.

## Conclusions

Overall, previously reported studies (*n =* 6) to prevent or treat obesity in Māori and Pacific Islander children and adolescents generated minimal impact in improving anthropometric indicators of weight or improving cardiometabolic or psychological secondary outcomes. There is a lack of evidence to recommend specific intervention characteristics that will optimise overweight/obesity prevention or treatment interventions in Māori and Pacific Islander children and adolescents. These results are possibly secondary to a lack of intervention intensity and specificity to Māori and Pacific Islander peoples. The authors propose the following recommendations for future research:

- Cultural-tailoring of interventions, preferably utilising a co-design approach, with adequate methodological reporting;
- Implementation of interventions that exclusively target Māori and Pacific Islander children and adolescents; fostering community engagement, leadership and ownership at every stage of the proposed intervention i.e. from conception to evaluation;
- Performing intervention sub-group analysis on Māori and Pacific Islander participants in mixed-population studies; and
- Integrating and evaluating, where possible, long-term, mixed-methods interventions within an existing healthcare system to maximise reach and sustainability for policy- and population-level impact

Consideration of these recommendations in future research will optimise interventions to tackle childhood overweight/obesity in the unique priority population of Māori and Pacific Islanders, who exhibit a significantly higher prevalence of childhood overweight/obesity in Australia and New Zealand, as well as demonstrate substantial socioeconomic and health disadvantage that inherently increases population risk for long-term overweight/obesity and its comorbidities.

## Supplementary information

**Additional file 1.** PRISMA checklist.docx

**Additional file 2.** Search strategy in EMBASE according to the PICO format.docx

**Additional file 3.** Individual quality assessment and risk of bias results for included studies.docx

## Data Availability

Not applicable.

## Abbreviations

PRISMA: Preferred Reporting Items for Systematic Reviews and Meta-Analyses
RCT: Randomised controlled trial
BMI: Body mass index
HR-QOL: Health-related quality of life
PA: Physical activity
HFH: Healthy Foods Hawaii
NZE: New Zealand European
BF: Body fat
SDS: Standard deviation score
BP: Blood pressure
HbA_1c_: Glycated haemoglobin
WHO: World Health Organisation
