# Supplementary material for: Interventions to prevent or treat childhood obesity in Māori & Pacific Islanders: a systematic review
Source: BMC Public Health. 2020 May 19;20:725. doi: 10.1186/s12889-020-08848-6 (PMC7236934; doi:10.1186/s12889-020-08848-6)
Supplement: Supplementary file 2 — Additional file 2. Search strategy in EMBASE according to the PICO format.docx [file 12889_2020_8848_MOESM2_ESM.docx]

| **Additional file 2:** Search strategy in Embase* according to the PICO format | |
| --- | --- |
| PICO | Design |
| Population | oceanic ancestry group'/exp OR  (New Zealand:ti,ab AND indigen*) OR Māori OR “pacific islanders” OR American Samoa:ti,ab OR Fiji:ti,ab OR Micronesia:ti,ab OR Palau:ti,ab OR Papua New Guinea:ti,ab OR Samoa:ti,ab OR “Solomon Islands”:ti,ab OR Timor-Leste:ti,ab OR Tonga:ti,ab OR Tuvalu:ti,ab OR Vanuatu:ti,ab  AND  Child* OR 'child'/exp OR Perinat*:ti,ab OR neonat*:ti,ab OR newborn*:ti,ab OR infan*:ti,ab OR bab*:ti,ab OR toddler*:ti,ab OR boy*:ti,ab OR girl*:ti,ab OR kid*:ti,ab OR juvenil*:ti,ab OR teen*:ti,ab OR pubescen*:ti,ab OR adolescen*:ti,ab OR child*:ti,ab OR 'pediatrics'/exp OR pediatric*:ti,ab OR paediatric*:ti,ab |
| Intervention | "Clinical Trial" OR "clinical trial, phase i" OR "clinical trial, phase ii" OR "clinical trial, phase iii" OR "clinical trial, phase iv" OR "controlled clinical trial" OR "multicenter study" OR "randomized controlled trial" OR 'clinical trial (topic)'/exp OR 'phase 1 clinical trial (topic)'/exp OR 'phase 2 clinical trial (topic)'/exp OR 'phase 3 clinical trial (topic)'/exp  OR 'controlled clinical trial (topic)'/exp OR 'randomized controlled trial (topic)'/exp OR 'multicenter study (topic)'/exp OR 'double blind procedure'/exp OR ((randomised:ti,ab OR randomized:ti,ab) OR (trial:ti,ab OR trials:ti,ab)) |
| Comparison | *No intervention or trial* |
| Outcome | 'obesity'/exp OR obese:ti,ab OR obesity:ti,ab OR 'body weight'/exp OR "body weight":ti,ab OR overweight:ti,ab OR "over weight":ti,ab OR 'body mass'/exp OR "body mass index":ti,ab OR BMI:ti,ab OR  "body fat":ti,ab OR “growth charts”:ti,ab |
| PICO, Population Intervention Comparison Outcome  *The search strategy was amended as appropriate for other databases | |
