# Supplementary material for: Interventions to prevent or treat childhood obesity in Māori & Pacific Islanders: a systematic review
Source: BMC Public Health. 2020 May 19;20:725. doi: 10.1186/s12889-020-08848-6 (PMC7236934; doi:10.1186/s12889-020-08848-6)
Supplement: Supplementary file 3 — Additional file 3. Individual quality assessment and risk of bias results for included studies.docx [file 12889_2020_8848_MOESM3_ESM.docx]

| **Additional file 3:** Individual quality assessment and risk of bias results for included studies in the present systematic review according to a modified Downs & Black checklist^(18)^ | | | | | | | |
| --- | --- | --- | --- | --- | --- | --- | --- |
| Question | Reporting | Anderson, C.Y *et al.* (2017)^(21)^ (NZ) | Chansavang, Y. *et al.* (2015)^(22)^ (NZ) | Gittelsohn, J. *et al.* (2010)^(23)^ (USA - Hawaii) | Maddison, R. *et al.* (2014)^(26)^ (NZ) | Rush, E. *et al.* (2012)^(25)^ (NZ) | Rush, R. *et al.* (2014)^(24)^ (NZ) |
| 1 | Hypothesis/aims/objective clearly described | 1 | 1 | 1 | 1 | 1 | 1 |
| 2 | Main outcomes in Introduction or Methods | 1 | 1 | 1 | 1 | 1 | 1 |
| 3 | Patient characteristics clearly described | 1 | 1 | 0 | 1 | 1 | 1 |
| 4 | Interventions of interest clearly described | 1 | 1 | 1 | 1 | 1 | 1 |
| 5 | Principal confounders clearly described | 2 | 0 | 2 | 2 | 2 | 2 |
| 6 | Main findings clearly described | 1 | 1 | 1 | 1 | 1 | 1 |
| 7 | Estimates of random variability provided for main outcomes | 1 | 1 | 1 | 1 | 1 | 1 |
| 8 | All adverse events of intervention reported | 0 | 0 | 0 | 0 | 0 | 0 |
| 9 | Characteristics of patients lost to follow-up described | 1 | 0 | 0 | 0 | 0 | 0 |
| 10 | Probability values reported for main outcomes | 1 | 1 | 1 | 1 | 1 | 0 |
| 11 | Subjects asked to participate were representative of source population | 0 | 0 | 0 | 0 | 1 | 1 |
| 12 | Subjects prepared to participate were representative of source population | 0 | 0 | 0 | 0 | 1 | 1 |
| 13 | Location and delivery of study treatment was representative of source population | 1 | 1 | 1 | 1 | 1 | 1 |
| 14 | Study participants blinded to treatment | 0 | 0 | 0 | 0 | 1 | 1 |
| 15 | Blinded outcome assessment | 0 | 0 | 0 | 0 | 0 | 0 |
| 16 | Any data dredging clearly described | 1 | 1 | 1 | 1 | 1 | 1 |
| 17 | Analysis adjust for differing lengths of follow up | 1 | 1 | 1 | 1 | 1 | 1 |
| 18 | Appropriate statistical tests performed | 1 | 1 | 1 | 1 | 1 | 1 |
| 19 | Compliance with interventions was reliable | 0 | 1 | 1 | 0 | 0 | 0 |
| 20 | Outcome measures were reliable and valid | 1 | 1 | 1 | 1 | 1 | 1 |
| 21 | All participants recruited from the same source population | 1 | 1 | 0 | 1 | 1 | 0 |
| 22 | All participants recruited over the same period | 1 | 1 | 0 | 1 | 1 | 0 |
| 23 | Participants randomized to treatment (s) | 1 | 0 | 0 | 1 | 1 | 1 |
| 24 | Allocation of treatment concealed from investigators and participants | 0 | 0 | 1 | 0 | 0 | 0 |
| 25 | Adequate adjustment for confounding | 1 | 0 | 1 | 1 | 1 | 1 |
| 26 | Losses to follow up taken into account | 1 | 1 | 0 | 0 | 1 | 1 |
| 27 | Did study perform power calculation?* | 0 | 0 | 1 | 1 | 1 | 0 |
| *Total* |  | 20 (Good) | 16 (Fair) | 17 (Fair) | 19 (Fair) | 23 (Good) | 19 (Fair) |
| *Modified according to Korakikis *et al.*^(19)^ | |  |  |  |  |  |  |
